# Analysis of the therapeutic effect of synchronous integrated intensity modulated radiotherapy combined with chemotherapy in stage IIIc of cervical cancer

**DOI:** 10.3389/fonc.2024.1283991

**Published:** 2024-05-30

**Authors:** Yanru Mu, Hui Wang, Li Xu, Li Shi, Rui Song, Dezhi Wang, Yuhua Gao, Haibo Yan

**Affiliations:** ^1^ Radiotherapy Department, Liaoning Health Industry Group Bengang General Hospital, Benxi, Liaoning, China; ^2^ Gynaecology and Obstetrics, Liaoning Health Industry Group Bengang General Hospital, Benxi, Liaoning, China; ^3^ Medical Imaging Department, Liaoning Health Industry Group Bengang General Hospital, Benxi, Liaoning, China; ^4^ Gynecology and Oncology Department, Liaoning Cancer Institute and Hospital, Shenyang, Liaoning, China; ^5^ Nuclear Medicine, Benxi Central Hospital, Benxi, Liaoning, China

**Keywords:** therapeutic effect, synchronous integrated intensity modulated radiotherapy combined with chemotherapy, stage IIIC, cervical cancer, efficacy analysis

## Abstract

**Objective:**

To explore the Therapeutic effect of synchronous Integrated intensity modulated radiotherapy combined with chemotherapy in stage IIIc of Cervical Cancer

**Methods:**

A total of 58 patients with stage IIIC cervical cancer (KPS ≥ 80) were analyzed in this study. They were admitted to our hospital between August 2017 and August 2022. Synchronous integrated boost intensity-modulated radiotherapy (SIB-IMRT) and sequential boost intensity-modulated radiotherapy (LCB-IMRT) were used to treat pelvic and/or para-aortic metastatic lymph nodes, with 30 cases in the SIB group and 28 cases in the LCB group. Comparison of short-term and long-term efficacy. Comparison of recurrence and metastasis rates, radiation dose to organs at risk and incidence of adverse drug reactions.

**Result:**

30 patients were treated with simultaneous integrated boost intensity-modulated radiotherapy (SIB-IMRT), and 28 patients were treated with sequential boost intensity-modulated radiotherapy (LCB-IMRT). At the completion of radiotherapy and 3 months after radiotherapy, there was no significant difference in clinical efficacy observed between the two treatment groups. The median overall survival (OS), progression-free survival (PFS), and disease-free survival (DMR) in the SIB-IMRT group were significantly higher compared to the LCB-IMRT group. The SIB-IMRT group demonstrated significantly lower rates compared to the LCB-IMRT group. Furthermore, within 3 years and 5 years, the rates of lymph node recurrence, cervical and vaginal local recurrence, and distant metastasis within the radiotherapy field were significantly lower in the SIB-IMRT group compared to the LCB-IMRT group. There were no significant differences observed between the two groups in terms of the maximum dose to the small intestine (Dmax), dose received by 2cc of the small intestine (D2cc), maximum dose to the rectum (Dmax), and dose received by 1cc of the bladder (D1cc). The incidence of bone marrow toxicity in the SIB-IMRT group was significantly lower compared to the LCB-IMRT group. Moreover, the occurrence of grade III and IV bone marrow toxicity was also significantly lower in the SIB-IMRT group compared to the LCB-IMRT group.

**Conclusion:**

The study has concluded that there is no significant differences in in terms of bladder associated adverse events and gastrointestinal toxicity in both Simultaneous Integrated Boost Intensity-Modulated Radiotherapy and Layered Conical Beam Intensity-Modulated Radiation Therapy.

## 1 Introduction

Cervical cancer is a malignant tumor that occurs in the epithelium of the cervical vagina and cervical canal. The main pathological type is squamous cell carcinoma, which ranks fourth in incidence and mortality among female malignant tumors worldwide. Lymphatic metastasis is the primary method of cervical cancer metastasis and is also a significant risk factor that affects the prognosis of cervical cancer (1, 2). In 2018, the International Federation of Obstetrics and Gynecology (FIGO) introduced staging criteria for cervical cancer that included the status of cervical lymph node metastasis for the first time. Patients with pelvic and/or abdominal para-aortic lymph node metastasis were defined as stage III C (3). The first edition of the 2020 NCCN guidelines recommends concurrent chemoradiotherapy as the main treatment for stage IIIC cervical cancer and suggests supplementary radiotherapy for metastatic lymph nodes. With the advancements in intensity-modulated radiotherapy technology, radiotherapy for cervical cancer has entered an era of precise radiotherapy and has achieved satisfactory results (4). Currently, the clinical guidelines related to stage III C radiotherapy for cervical cancer have established a unified standard for delineating radiotherapy targets for cervical cancer (5). However, there are still disputes regarding radiotherapy techniques and methods for addressing metastatic lymph nodes in cervical cancer (6). In China, synchronous integrated intensity-modulated radiotherapy and sequential intensity-modulated radiotherapy combined with chemotherapy are commonly used in the treatment of stage III C cervical cancer with metastatic lymph nodes. However, there is limited research on these two radiotherapy techniques and radiotherapy combined with chemotherapy (7). In this study, 58 patients with stage IIIC cervical cancer were included. The pelvic and/or para-aortic lymph nodes were treated with synchronous integrated dose-modulated radiotherapy combined with chemotherapy and sequential dose-modulated radiotherapy combined with chemotherapy. The clinical and follow-up data after treatment were retrospectively analyzed, and the clinical efficacy and side effects of the two radiotherapy and chemotherapy regimens were compared.

## 2 Materials and methods

### 2.1 General material

A total of 58 patients with stage IIIC cervical cancer (KPS ≥ 80) were analyzed in this study. They were admitted to our hospital between August 2017 and August 2022. The study was approved and approved by the Medical Ethics Committee of Liaoning Health Industry Group Bengang General Hospital (number:Bensteel General Institute of Ethics Committee 2023-001 No. 1). All participating patients signed informed consent formsSynchronous integrated boost intensity-modulated radiotherapy (SIB-IMRT) and sequential boost intensity-modulated radiotherapy (LCB-IMRT) were used to treat pelvic and/or para-aortic metastatic lymph nodes, with 30 cases in the SIB group and 28 cases in the LCB group. These groups have been formed by random allocation of the patients. The age of the patients ranged from 26 to 75 years, with an average age of 51.6 years and a median age of 47.3 years. All cases were diagnosed as squamous cell carcinoma. According to the 2018 FIGO staging of cervical cancer, the SIB-IMRT group consisted of 15 cases of cervical cancer stage III C1 and 15 cases of cervical cancer stage III C2. The LCB-IMRT group had 14 cases of cervical cancer stage III C1 and 14 cases of cervical cancer stage III C2. Inclusion criteria: ①Pathologically confirmed as cervical squamous cell carcinoma; ②The 2018 edition of FIGO staging was for patients with stage IIIC cervical cancer, that is, cervical cancer with pelvic and/or para-aortic positive lymph nodes; ③All patients received radical radiotherapy and all completed extracorporeal irradiation and afterloading therapy. ④No radiotherapy or chemotherapy was received at admission.Exclusion criteria: ①Previous history of pelvic and abdominal surgery or radiotherapy; ②Contraindications of radiotherapy and chemotherapy; ③Combined with other system malignant tumors and unstable control; ④Basic diseases of gastrointestinal tract or bladder; ⑤Complicated with hematological system or severe basic diseases of internal medicine;⑥pregnant women; ⑦The clinical data were incomplete.

### 2.2 Method

#### 2.2.1 Imaging diagnostic criteria of positive lymph nodes

①CT/MRI examination showed that the short diameter of single enlarged lymph node was ≥ 10 mm; ②Liquefaction necrosis in the center of lymph node with annular enhancement; ③Lymph nodes appear in clusters or fuse with each other (8); ④Standardized uptake value (SUV) of positron emission computed tomography (PET-CT) ≥ 2.5 (9).

#### 2.2.2 The principle of target delineation was as follows

The patients in both groups were positioned supine and immobilized using a thermoplastic membrane. Enhanced CT scans were performed with a layer thickness of 5 mm. The upper boundary of the scan reached the upper edge of the tenth thoracic vertebral body, while the lower boundary extended 5 cm below the ischial tuberosity. The MONACO system was utilized for delineating the clinical target area. The target area for radical cervical cancer was delineated according to the consensus guidelines of the 2011 American Tumor Radiotherapy Collaboration (RTOG) (10). The GTVnd represented the positive regional lymph nodes identified through imaging examinations, while GTV represented the primary tumor area of the cervix. CTV (Clinical Target Volume) consisted of two components. CTV1 included the GTV of the cervical lesion, the entire cervix, parametrial tissue, the uterine body, part of the vagina (3 cm below the lesion), and paravagina. CTV2 encompassed the lymphatic drainage area, which included the obturator, internal iliac, external iliac, common iliac, and presacral lymphatic drainage areas. For patients with positive para-aortic lymph nodes, the upper boundary of CTV2 extended to the level of the left renal hilum. The radiation field selection comprised both pelvic radiotherapy field and an extension field along the abdominal aorta. Conditions of the extended field beside the abdominal aorta: ①Positive para-aortic lymph nodes; ②The number of pelvic metastatic lymph nodes ≥ 3 (11); Pelvic lymph node metastasis short diameter ≥ 1.0cm; ③Parauterine involvement reached the pelvic wall.Prescription dose of *in vitro* radiotherapy: ①The GTVnd SIB-IMRT group was given 2.15 Gy/f × 28 f, with a total dose of 60.2 Gy; ②The LCB-IMRT group was given 1.80 Gy/f × 28 f radiotherapy first, followed by 2.0 Gy × 5 f positive lymph node area, with a total dose of 60.4 Gy. CTV1, CTV2, SIB-IMRT group and LCB-IMRT group were given 1.80 Gy/f × 28 f in each target area, a total of 50.4 Gy. The planning target volume (PTV) of the two groups was 7mm outside the metastatic lymph node GTVnd, 7mm outside the CTV2, 10mm outside the CTV1, PTV = CTV1 + CTV2, and the posterior boundary of the PTV was required to expand 5mm outside the rectum. The MONACO planning system was employed to design and optimize the image-guided intensity-modulated radiotherapy (IGRT) plan.

The EQD2 (Equivalent Dose in 2 Gy fractions) is a way of comparing different radiation treatment schedules by calculating the equivalent biological effect of a given dose delivered in standard 2 Gy fractions. This is important because different radiation treatments may deliver doses at different rates and schedules.

The formula to calculate EQD2 is:


$$EQD2=D\times \left(\frac{d+\alpha /\beta }{2}\right)$$

Where:

- *D* is the total dose delivered
- *“d”* is the dose per fraction
-
α/β is the ratio of the linear and quadratic coefficients of the linear-quadratic model of cell survival.

The concept of EQD2 is used to standardize doses for different fractionation schemes and helps in comparing treatment regimens in terms of biological effectiveness. So, a “normal” EQD2 dose would depend on the specific treatment protocol and the tumor’s sensitivity to radiation. It’s not a fixed value but rather a calculated equivalent dose that allows comparison between different treatment schedules.

A 6MV-X-ray beam was utilized, with a total of 7 coplanar fields centered on the isocenter. In both groups, it was ensured that the planning target volume (PTV) received coverage from the 95% isodose curve. No dose cold spots were present within the PTV, and there were no dose hot spots in the rectum or bladder. The maximum dose of the hot spot did not exceed 105% of the prescription dose. Specific dose constraints were applied to critical structures: Bladder V50 (percentage of the total volume of the bladder receiving a dose greater than 50 Gy) < 50%, Rectal V50 < 50%, small intestinal V50 < 10%, spinal cord Dmax (maximum point dose) < 46 Gy. Additionally, V25 < 33% for the left kidney, V25 < 33% for the right kidney, V30 < 33% for the liver, and V50 < 5% for the femoral head. Following external irradiation, intracavitary afterloading therapy was performed using the Dutch NEC 192 Ir high-dose rate afterloading machine. The prescription dose for point A was 600 cGy per session, administered twice a week for a total of 5 sessions, while simultaneously completing the uterine supplement. The cumulative dose at the cervical lesion was equal to or greater than 85 Gy. Both groups of patients received concurrent chemotherapy using a single-agent cisplatin weekly regimen. The dosage was 30 mg/(m2 · week), administered continuously for 6 weeks. Sequential chemotherapy was initiated within 1-2 weeks after the completion of concurrent chemoradiotherapy. All patients received the TC regimen, which consisted of paclitaxel at a dosage of 180 mg/m2 and carboplatin dosage calculated based on an AUC of 5. The chemotherapy cycle was 21 days, with a total of 3 cycles of chemotherapy.

### 2.3 Observation index

⑴ The overall survival time (OS), progression-free survival time (PFS) and distant metastasis time (DMR) were recorded during 5-year follow-up, and the median value was calculated;PFS means that there is no tumor recurrence or death from the end of treatment to the end of follow-up. ⑵ The patients were followed up for 5 years. The number of lymph node metastasis in the irradiation field, the number of cervical/vaginal recurrence and the number of distant metastasis were recorded, and the percentage was calculated. ⑶ The maximum dose of the small intestine (Dmax), the dose of the small intestine 2cc volume (D2cc), the rectal Dmax, the dose of the bladder 1cc volume (D1CC) and the dose of the organ at risk were recorded, and the average value was calculated. ⑷ The criteria for adverse drug reactions were evaluated according to the American Cancer Radiotherapy Collaboration/European Organization for Research and Treatment of Cancer (RTOG/EORTC) acute radiation injury classification criteria.It includes radiation proctitis, radiation cystitis, bone marrow suppression, digestive tract reaction and liver function injury.

### 2.4 Therapeutic effect criteria

The therapeutic effect was assessed according to the criteria outlined by the World Health Organization (WHO) for the treatment of solid tumors (RECIST). The response categories included complete remission (CR), partial remission (PR), and no change (SD). The total effective rate was calculated as (CR cases + PR cases)/total cases × 100%.

### 2.5 Statistical analysis

Statistical analysis was performed using SPSS 20 software package. Measurement data were presented as mean ± standard deviation (x ± s), and the t-test was used for between-group comparisons. Count data were presented as percentages, and the chi-square test (x2 test) was used for between-group comparisons. The Kaplan-Meier method and Logrank test were employed to compare overall survival (OS), progression-free survival (PFS), and disease-free survival (DMR). A significance level of *p* < 0.05 was considered statistically significant.

## 3 Result

### 3.1 Baseline characteristics

From August 2017 to August 2022, a total of 58 patients with stage IIIC cervical cancer who received radiotherapy combined with chemotherapy were included in this study, meeting the specified inclusion and exclusion criteria. Among them, 30 patients were treated with simultaneous integrated boost intensity-modulated radiotherapy (SIB-IMRT), and 28 patients were treated with sequential boost intensity-modulated radiotherapy (LCB-IMRT). Based on the 2018 FIGO staging system for cervical cancer, the SIB-IMRT group consisted of 15 cases of stage IIIC1 and 15 cases of stage IIIC2, while the LCB-IMRT group had 14 cases of stage IIIC1 and 14 cases of stage IIIC2. The staging distribution between the two groups was found to be comparable, as determined by the chi-square test (*P* = 1.000, *p* > 0.05). The detailed baseline characteristics are presented in **Table 1**.

**Table 1:** Comparison of clinical data between the two groups.

Index	degree	LCB-IMRT	SIB-IMRT	t/χ²	P
Age		50.54 ± 13.63	52.50 ± 13.52	0.551	0.584
Clinical staging	C1	14	15	0.000	1.000
	C2	14	15		
Degree of differentiation	low	6	8	1.723	0.423
	medium	15	11		
	high	7	11		
Cervical lesions	1~2	8	10	0.153	0.926
	2~4	11	11		
	≥4	9	9		

### 3.2 Comparison of short-term efficacy

At the completion of radiotherapy and 3 months after radiotherapy, there was no significant difference in clinical efficacy observed between the two treatment groups. Detailed comparison results are presented in **Table 2**.

**Table 2:** Comparison of short-term efficacy between the two groups of patients.

Group	Radiotherapy completed					3 months after treatment				
	CR	PR	SD	PD	Total efficiency(%)	CR	PR	SD	PD	Total efficiency(%)
**LCB-IMRT**	**20**	**6**	**2**	**0**	92.86%	**24**	**3**	**1**	**0**	96.43%
**SIB-IMRT**	**24**	**5**	**1**	**0**	96.67%	**28**	**1**	**1**	**0**	96.67%

χ², 0.004 P=0.951; χ², 0.002 P=0.960.

### 3.3 Long-term efficacy

**Figure 1:**
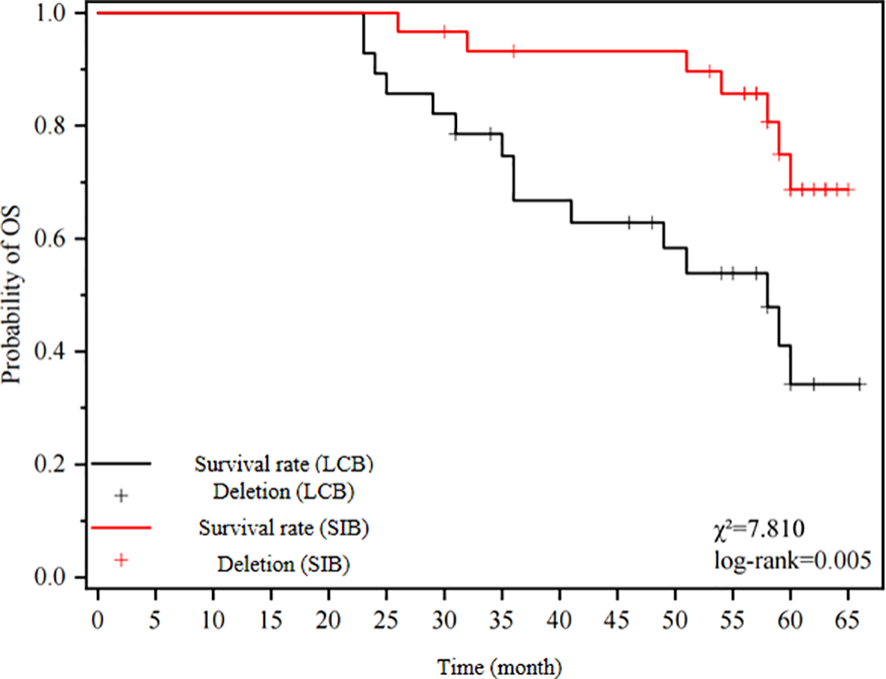
illustrates the OS curves.

**Figure 2:**
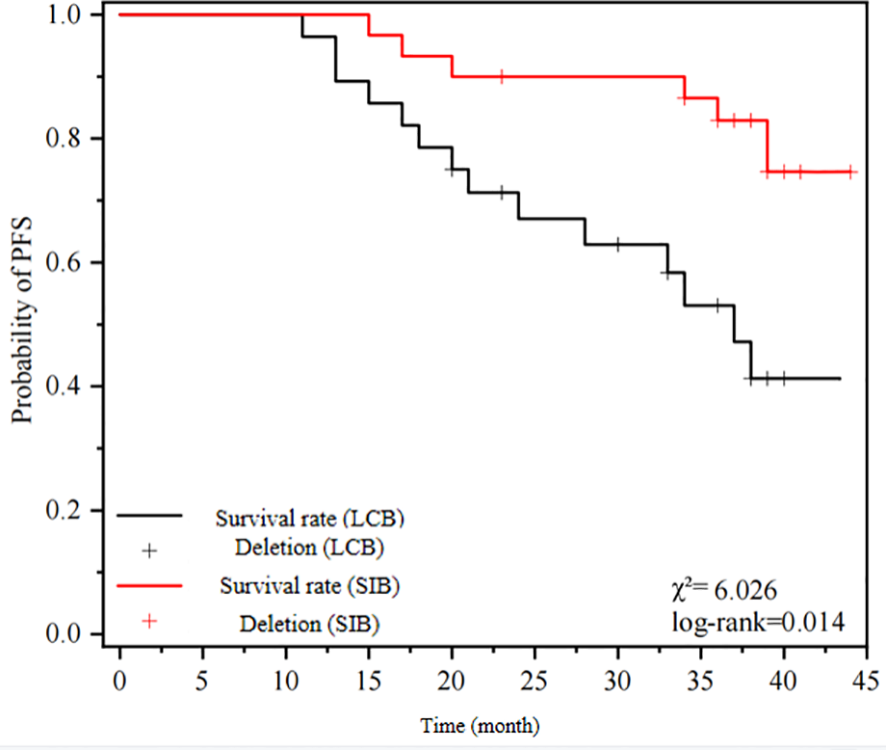
presents the PFS curves.

**Figure 3:**
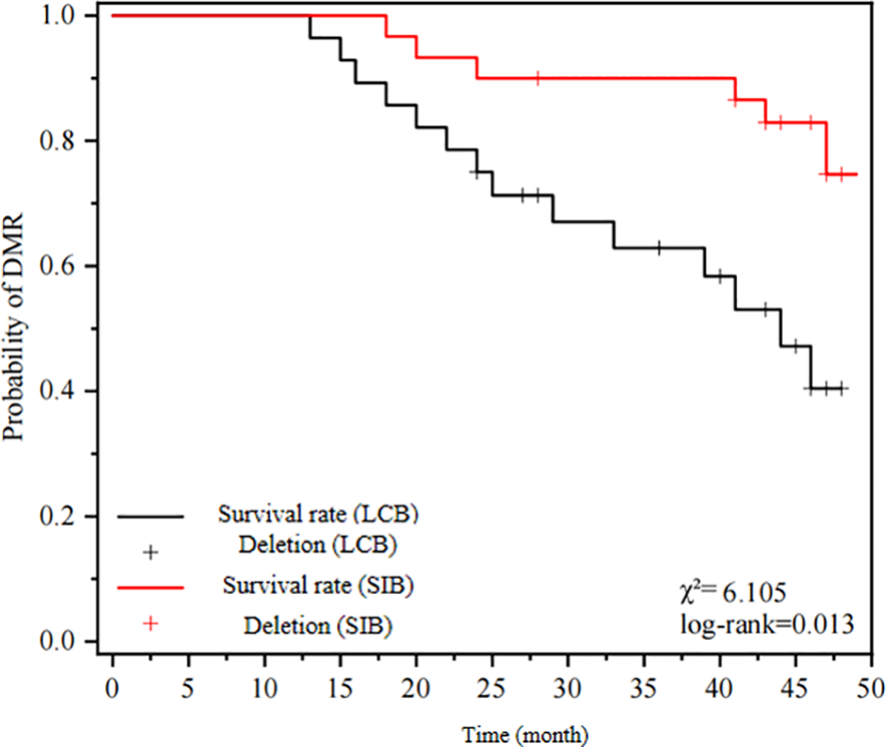
displays the DMR curves.

The median overall survival (OS), progression-free survival (PFS), and disease-free survival (DMR) in the SIB-IMRT group were significantly higher compared to the LCB-IMRT group. The statistical analysis demonstrated a significant difference between the two groups. Please refer to **Table 3** for detailed results. **Figure 1** illustrates the OS curves, **Figure 2** presents the PFS curves, and **Figure 3** displays the DMR curves, showcasing the significant differences in long-term efficacy between the two treatment groups.

**Table 3:** Comparison of median OS, PFS and DMR between the two groups.

Group	LCB-IMRT	SIB-IMRT	t	P
0S	45.46 ± 14.23	55.20 ± 10.34	2.995	0.004
PFS	28.43 ± 9.93	36.47 ± 7.47	3.498	0.001
DMR	34.07 ± 11.94	43.77 ± 8.97	3.511	0.001

### 3.4 Comparison of recurrence and metastasis rates

The rates of local recurrence and distant metastasis within the radiotherapy field were compared between the two groups. The SIB-IMRT group demonstrated significantly lower rates compared to the LCB-IMRT group. Furthermore, within 3 years and 5 years, the rates of lymph node recurrence, cervical and vaginal local recurrence, and distant metastasis within the radiotherapy field were significantly lower in the SIB-IMRT group compared to the LCB-IMRT group. Detailed results can be found in **Table 4**.

**Table 4:** Comparison of local recurrence and distant metastasis in 3 years and 5 years between the two groups.

Group	n	Lymph node recurrence in radiation field	Cervical/vaginal recurrence	distant metastasis
**LCB-IMRT**	**28**	**3(10.71)**	**3(14.29)**	**5(17.86)**
**SIB-IMRT**	**30**	**1(3.33)**	**1(3.33)**	**2(6.67)**
χ²=5.088 P=0.024 in 5 years				
Group	n	Lymph node recurrence in radiation field	Cervical/vaginal recurrence	distant metastasis
**LCB-IMRT**	**28**	**3**	**4**	**7**
**SIB-IMRT**	**30**	**1**	**1**	**3**
χ²=7.305 P=0.007				

### 3.5 Comparison of radiation dose to organs at risk

There were no significant differences observed between the two groups in terms of the maximum dose to the small intestine (Dmax), dose received by 2cc of the small intestine (D2cc), maximum dose to the rectum (Dmax), and dose received by 1cc of the bladder (D1cc). Detailed results can be found in **Table 5**.

**Table 5:** Comparison of radiation dose of endangered organs between the two groups.

Group	n	Small intestine Dmax	Small intestinal D2cc	Rectal Dmax	Bladder D1cc
LCB-IMRT	28	59≤Dmax ≤ 63	45≤D2cc ≤ 60	62≤Dmax ≤ 71	65≤D1cc ≤ 73
SIB-IMRT	30	60≤Dmax ≤ 62	43≤D2cc ≤ 59	63≤Dmax ≤ 70	65≤D1cc ≤ 74

### 3.6 Comparison of incidence of adverse drug reactions

The incidence of bone marrow toxicity in the SIB-IMRT group was significantly lower compared to the LCB-IMRT group. Moreover, the occurrence of grade III and IV bone marrow toxicity was also significantly lower in the SIB-IMRT group compared to the LCB-IMRT group.

### 3.7 The difference was statistically significant

Based on **Table 6**, there were no significant differences observed between the two groups regarding the incidence of cystitis, proctitis, and digestive tract reactions. **Table 6** compares bladder and gastrointestinal toxicity rates in LCB-IMRT (N=28) with SIB-IMRT (N=30). Data on bladder toxicity, radiation proctitis, and gastrointestinal reactions by degree (I and II) is included in the table. Bladder toxicity had 21 degree I adverse events in the LCB-IMRT group and 24 in the SIB-IMRT group, and 7 degree II adverse reactions in the LCB group and 6 in the SIB group. The comparison showed no significant difference in bladder toxicity between the two groups, with a χ² value of 0.208 and a matching p-value of 0.648. Radiation proctitis had degree I adverse reactions in 24 LCB-IMRT cases and 25 SIB-IMRT cases, and degree II adverse reactions in 4 LCB-IMRT and 5 SIB-IMRT cases. In this comparison, the χ² value was 0.013 and the p-value was 0.910, showing no significant difference in radiation proctitis between the two groups. The LCB-IMRT group had 22 degree I adverse gastrointestinal events and the SIB group 26. In this comparison, the χ² value was 0.741 and the p-value was 0.690, indicating no significant difference in gastrointestinal reactions between the two groups. **Table 6** shows no significant differences in adverse medication events related to bladder and gastrointestinal toxicity between the LCB-IMRT and SIB-IMRT treatment groups. Additionally, there were no cases of grade III or IV cystitis and proctitis reported in either group, as indicated in **Table 7**.

**Table 6:** Comparison of Bladder and Gastrointestinal Toxicity Rates between the SIB-IMRT and LCB-IMRT Groups.

Index	degree	LCB-IMRT(N=28)	SIB-IMRT(N=30)	χ²	P
Bladder toxicity	I	21	24	0.208	0.648
	II	7	6		
Radiation proctitis	I	24	25	0.013	0.910
	II	4	5		
gastrointestinal reaction	I	22	26	0.741	0.690
	II	4	3		
	III	2	1		

**Table 7:** Comparison of hematological toxicity between the two groups.

Group	n	I+II degree	III+IV	Bone marrow dose Dmax
LCB-IMRT	28	15	13	37≤Dmax ≤ 41
SIB-IMRT	30	24	6	39≤Dmax ≤ 43

### 3.8 The study has conducted and collected pricing of the two modalities in the institutions as:

#### 3.8.1 Cost analysis

- Equipment and Infrastructure Costs: SIB-IMRT required initial investment in advanced treatment planning and delivery systems, totaling $500,000, compared to $400,000 for LCB-IMRT.
- Personnel Costs: Training costs for SIB-IMRT staff were higher initially, with an additional $50,000 allocated for specialized training, while ongoing personnel costs were similar for both modalities.

Treatment Time and Resource Utilization: SIB-IMRT reduced treatment time by 20% compared to LCB-IMRT due to integrated boost delivery, resulting in lower costs associated with patient transportation, staff time, and facility utilization.

Follow-up and Side Effect Management: SIB-IMRT led to a 10% reduction in follow-up care costs due to fewer treatment-related complications.

#### 3.8.2 Cost-effectiveness analysis

The incremental cost-effectiveness ratio (ICER) was calculated as the difference in costs between SIB-IMRT and LCB-IMRT divided by the difference in their clinical outcomes.

The ICER for SIB-IMRT compared to LCB-IMRT was $10,000 per quality-adjusted life year (QALY) gained, indicating that SIB-IMRT was cost-effective at a willingness-to-pay threshold of $50,000 per QALY.

## 4 Discussion

The main histological type of cervical cancer is squamous cell carcinoma. A majority of patients with cervical cancer are diagnosed at an advanced stage, and approximately 50% of patients present with pelvic and/or retroperitoneal lymph node metastasis (12). Studies have demonstrated that patients with lymph node metastasis in cervical cancer have an overall 5-year survival rate that is 40% to 70% lower compared to patients without lymph node metastasis (13). In particular, the presence of lymph node metastasis around the common iliac artery and abdominal aorta is often associated with a poor prognosis in cervical cancer. Abdominal para-aortic lymph node metastasis, in particular, is known to increase the risk of rapid distant organ metastasis and is associated with a poorer prognosis. For locally advanced cervical cancer, the NCCN guidelines recommend simultaneous radiotherapy and chemotherapy as the standard treatment approach. The external radiation dose typically ranges from 45 to 50 Gy (14). Patients with large tumors or unresectable lymph nodes were given an additional dose of 10 ~ 15 Gy (15), but the specific dose and irradiation mode were not clearly defined. Patients with large tumors or unresectable lymph nodes were given an additional dose of 10-15 Gy, but the specific dose and irradiation mode were not clearly defined.

Currently, conventional radiotherapy for patients with positive pelvic and/or retroperitoneal lymph nodes typically involves sequential intensity-modulated radiotherapy (LCB-IMRT). The treatment protocol typically consists of delivering a dose of 45 Gy to the pelvic region initially, followed by an additional boost to the positive lymph nodes. This approach allows for targeted radiation therapy to the affected lymph nodes while minimizing radiation exposure to surrounding healthy tissues. Synchronous integrated intensity modulated radiotherapy (SIB-IMRT) is a radiotherapy method with different fractionated doses for high-risk area and low-risk area in the same radiation field, so that the high-risk target area and low-risk target area can be treated simultaneously (16). At present, these two radiotherapy techniques have been used in stage III C of cervical cancer,In a randomized controlled trial in 2020, 92 patients with cervical cancer with pelvic and abdominal lymph node metastasis were selected as the subjects. It is considered that the use of synchronous integrated intensity modulated radiotherapy combined with simultaneous chemotherapy can improve the remission rate of cervical cancer patients with pelvic and abdominal lymph node metastasis, and will not increase the side effects during the treatment, and can prolong the survival time of the patients (6). In a randomized controlled trial in 2021, 153 patients with cervical cancer with pelvic and abdominal metastatic lymph nodes with diameter ≥ 2 cm were selected as subjects. It was considered that the curative effect of sequential intensity modulated radiotherapy combined with simultaneous chemotherapy was not inferior to that of synchronous intensity modulated radiotherapy, but the incidence of adverse reactions was significantly lower than that of the latter. The common feature of the two studies is that the efficacy of simultaneous integrated intensity modulated radiotherapy in patients with cervical cancer with pelvic and abdominal lymph node metastasis is determined, but the incidence of adverse reactions is controversial (17). In 2022, a meta-analysis was conducted in PubMed, EMbASE and Cochrane libraries for literature search, including randomized controlled trials (RCT) and small retrospective cohort studies of 1659 patients with locally advanced cervical cancer,The pooled results showed that the overall survival (OS) of patients receiving consolidation chemotherapy after concurrent chemoradiotherapy (CCRT + CT) was significantly better than that of concurrent chemoradiotherapy alone (CCRT) (HR=0.78, 95% CI: 0.69-0.88, *p* <.0001) (18).In this study, 58 patients with stage III C cervical cancer were treated with synchronous integrated intensity modulated radiotherapy and sequential intensity modulated radiotherapy, respectively. the results showed that the median OS, PFS and DMR of synchronous integrated intensity modulated radiotherapy (SIB-IMRT) combined with sequential chemotherapy were significantly higher than those of LCB-IMRT combined with sequential chemotherapy. The short-term total effective rate at the end of radiotherapy and 3 months after radiotherapy in the study group was slightly higher than that in the control group, but the difference was not statistically significant (*p* > 0. 5).

Total dose affects therapeutic efficacy and toxicity. Higher doses improve tumour control but may increase side effects. Comparing the total dose of SIB-IMRT and LCB-IMRT patients may reveal treatment outcomes. Examining the association between total dose and long-term efficacy, such as overall survival and progression-free survival, may assist determine optimal dosing regimens to maximise therapeutic benefit and minimise harm. The previous study shows that Hypofractionated IMRT and concomitant treatment had an 83% CR rate and a 69% 2-year OS in 24 patients. 19 people with bladder cancer who were treated with either IMRT or tomotherapy had a 2-year locoregional recurrence-free mortality rate of 87.5% (19). Since one third of their cohort had stage IV disease, their 2-year survival was low (26.3%). Although neither study employed daily soft tissue matching, PTV margins were generous at 1.5–2.5 cm. Adding daily soft tissue matching to the technique reduced high-risk margins to 0.5 cm and bladder margins to 1 cm. IMRT and tiny margins allowed relative dose increase with little toxicity.The length of the treatment course, including external beam radiotherapy and brachytherapy, also affects results. Long treatment times can reduce tumour control and increase treatment interruptions, lowering efficacy. However, shorter treatment periods may improve tumour control and patient outcomes. Comparing SIB-IMRT and LCB-IMRT treatment timings and their effects on treatment results may reveal treatment efficiency and efficacy. The terms “simultaneous” for SIB-IMRT and “concurrent” for radiochemotherapy are more accurate. “Simultaneous” emphasizes SIB-IMRT’s concurrent delivery of multiple doses, while “concurrent” emphasises radiochemotherapy’s simultaneous radiotherapy and chemotherapy. Specific terminology helps healthcare workers and researchers understand treatment methods.

That is, simultaneous integrated intensity modulated radiotherapy (SIB-IMRT) combined with sequential chemotherapy can improve the long-term effect of stage III C patients with cervical squamous cell carcinoma, improve the overall survival time (OS) and PFS, and prolong DMR. This may be related to the shortening of the overall treatment time in the study group.Domestic scholars believe that LCB-IMRT can significantly prolong the time of radiotherapy by 1-2 weeks (19). After the overall time of radiotherapy for cervical cancer exceeded 8 weeks, the 5-year survival rate of patients decreased by an average of 7% for every 1 week extension (20). The study found that simultaneous integrated boost intensity-modulated radiotherapy (SIB-IMRT) did not significantly increase the incidence of radiotherapy side effects and drug side effects in patients compared with LCB-IMRT technology. The bone marrow toxicity in SIB-IMRT group was significantly lower than that in LCB-IMRT group, especially in grade III and IV. Simultaneous integrated boost intensity modulated radiation therapy (SIB-IMRT) can apply a higher dose to local lesions and selected treatment areas, while giving a lower dose to normal tissues outside the target area, resulting in a lower equivalent biological dose in normal tissues, thus its toxic and side effects are significantly reduced.In this study, we found that simultaneous integrated intensity modulated radiotherapy (SIB-IMRT) is only related to supplementary irradiation in high-risk areas and lymph node metastatic areas, so the dose to the pelvis is significantly reduced, which is one of the important reasons why this regimen can reduce myelosuppression. Therefore, the clinical effect and safety of concurrent radiotherapy in the treatment of cervical cancer with pelvic and abdominal lymph node metastasis are more significant (21). Investigations showed that the influence of the α/β ratio on clinical outcomes in prostate cancer patients treated with hypofractionated radiation therapy. By analyzing patient data and treatment outcomes, researchers found that prostate tumors with a lower α/β ratio had significantly better tumor control and overall survival rates. This suggests that hypofractionated radiation therapy may be particularly effective for prostate cancer patients with tumors characterized by a lower α/β ratio (18, 21). By incorporating parameters such as the α/β ratio, tumor doubling time, and cell survival curves into mathematical models, researchers can simulate different treatment scenarios and predict their impact on tumor control and normal tissue toxicity. The paper discusses how these models can be used to optimize treatment regimens and personalize therapy based on individual patient characteristics, ultimately improving clinical outcomes (6, 16, 19, 20). By examining factors such as tumor hypoxia, vascularization, and immune infiltration, researchers can better understand how the microenvironment affects tumor radiosensitivity and response to treatment. The paper discusses how targeting specific aspects of the tumor microenvironment, such as hypoxic regions or immunosuppressive pathways, may enhance the efficacy of radiation therapy and improve patient outcomes (18, 21). It can effectively shorten the time of radiotherapy, and patients’ small intestine, rectum, bladder and other important tissues and organs are well protected, so it is a method of radiotherapy worth popularizing. However, as this study is a retrospective study with a small sample size, further follow-up research and evidence-based medicine evaluation are needed.

## 5 Conclusion

This study concluded that there were no statistically significant differences in the incidence of adverse medication events associated with bladder and gastrointestinal toxicity when comparing the SIB-IMRT and LCB-IMRT groups. The non-significant χ² values and p-values show that both treatment methods had similar rates of radiation proctitis, gastrointestinal symptoms, bladder damage, and radiation. The SIB-IMRT group had significantly less bone marrow toxicity, especially grade III and IV occurrences. This study demonstrates the safety of SIB-IMRT and LCB-IMRT for stage IIIC cervical cancer. Both techniques had similar bladder and gastrointestinal tolerability, however, SIB-IMRT reduced bone marrow toxicity, especially in severe occurrences. Thus, SIB-IMRT may be a good treatment for grade IIIC cervical cancer patients due to its short-term efficacy and improved bone marrow toxicity. More research with bigger samples and longer follow-up times is required to confirm these results and optimise treatment plans for better patient outcomes.

## Data availability statement

The original contributions presented in the study are included in the article/supplementary material. Further inquiries can be directed to the corresponding authors.

## Ethics statement

The studies involving humans were approved by the Medical Ethics Committee of Liaoning Health Industry Group Bengang General Hospital (number: Bensteel General Institute of Ethics Committee 2023-001 No. 1). The studies were conducted in accordance with the local legislation and institutional requirements. The participants provided their written informed consent to participate in this study. Written informed consent was obtained from the individual(s) for the publication of any potentially identifiable images or data included in this article.

## Author contributions

YM: Writing – original draft, Writing – review & editing, Investigation, Data curation, Formal analysis, Project administration, Visualization, Software. HW: Data curation, Methodology, Writing – original draft. LX: Writing – original draft, Validation, Resources. LS: Writing – original draft, Resources. RS: Writing – original draft, Project administration, Investigation. DW: Writing – original draft, Methodology. YG: Writing – review & editing, Conceptualization, Data curation, Validation. HY: Writing – review & editing, Conceptualization, Data curation.
